# Timing constraints of action potential evoked Ca^2+^ current and transmitter release at a central nerve terminal

**DOI:** 10.1038/s41598-019-41120-5

**Published:** 2019-03-14

**Authors:** Owen Y. Chao, Yi-Mei Yang

**Affiliations:** Department of Biomedical Sciences, University of Minnesota Medical School, 1035 University Drive, Duluth, MN 55812 USA

## Abstract

The waveform of presynaptic action potentials (APs) regulates the magnitude of Ca^2+^ currents (I_Ca_) and neurotransmitter release. However, how APs control the timing of synaptic transmission remains unclear. Using the calyx of Held synapse, we find that Na^+^ and K^+^ channels affect the timing by changing the AP waveform. Specifically, the onset of I_Ca_ depends on the repolarization but not depolarization rate of APs, being near the end of repolarization phase for narrow APs and advancing to the early repolarization phase for wide APs. Increasing AP amplitude has little effect on the activation but delays the peak time of I_Ca_. Raising extracellular Ca^2+^ concentration increases the amplitude of I_Ca_ yet does not alter their onset timing. Developmental shortening of APs ensures I_Ca_ as a tail current and faithful synaptic delay, which is particularly important at the physiological temperature (35 °C) as I_Ca_ evoked by broad pseudo-APs can occur in the depolarization phase. The early onset of I_Ca_ is more prominent at 35 °C than at 22 °C, likely resulting from a temperature-dependent shift in the activation threshold and accelerated gating kinetics of Ca^2+^ channels. These results suggest that the timing of Ca^2+^ influx depends on the AP waveform dictated by voltage-gated channels and temperature.

## Introduction

Classic work on invertebrate synapses from squid, crayfish and *Aplysia* demonstrate that the waveform of presynaptic action potentials (APs) regulates Ca^2+^ influx through voltage-gated Ca^2+^ channels (VGCCs) into nerve terminals, and ultimately synaptic strength and temporal fidelity^1–6^. Recent studies in mammalian central synapses, with simultaneous optical imaging of voltage-sensitive and Ca^2+^ indicators or direct patching of large nerve terminals of model synapses, have gained significant insights into intricate interplays between the AP waveform, Ca^2+^ influx and quantal output^7–11^. These studies have yielded general agreements on the properties of Ca^2+^-dependent transmitter release with those from invertebrates, but also revealed fundamental differences, for example, in the fraction of VGCCs activated by a single AP and the cooperative nature of activated VGCCs in triggering release events^11–14^. Although these reports have provided quantitative descriptions of the biophysical behaviors of VGCCs in response to an AP and the downstream coupling of Ca^2+^ influx to vesicular release, how an AP constrains the timing of presynaptic Ca^2+^ influx and transmitter release remains controversial from studies on different central synapses^7–9^. At the cerebellar granule cell-stellate cell synapses, Ca^2+^ influx appears to occur during the depolarization phase of an AP (*i*.*e*. on currents) and is strongly temperature-dependent^7,15^. On the contrary, Ca^2+^ entry at the immature calyx of Held and mossy fiber-CA3 synapses always coincides with the repolarization phase (*i*.*e*. off or tail currents)^8,9,16^, independent of temperature^8^. Similar composition of VGCCs (mainly high voltage-gated N- and P/Q-types) at these synapses^17–19^ raises the possibility that distinct phenotypes in the timing control may be accounted for by upstream elements that dictate Ca^2+^ inflow through these channels, such as the waveform of APs and other experimental conditions including extracellular Ca^2+^ concentration and temperature.

Unlike the invertebrate neurons, mammalian central nervous system employs a large variety of Na^+^ and K^+^ channels, giving rise to diverse shapes of APs^20^. For example, fast-spiking neurons usually elicit much narrower APs than slow-spiking neurons due to abundance of high-threshold K^+^ channels^21^. Even within a same neuron, APs can appear very differently in the soma and nerve terminals because of non-uniform distribution of voltage-gated ion channels^9^. Furthermore, APs undergo changes in the amplitude and width during development and repetitive neural activity resulting from inactivation or facilitation of Na^+^ and K^+^ channels^9,22–28^.

To systematically study how the diverse waveform of presynaptic APs determines the timing of Ca^2+^ influx and transmitter release, we have performed voltage clamp recordings of presynaptic calcium currents (I_Ca_) and excitatory postsynaptic currents (EPSC) at the calyx of Held synapse in the mouse auditory brainstem, which is an ideal model for biophysical analysis of synaptic properties^12,14,29–34^. By blocking presynaptic voltage-gated K^+^ and Na^+^ channels with tetraethylammonium (TEA) and tetrodotoxin (TTX) respectively, we find that both channels contribute to the onset timing of I_Ca_ and EPSC by targeting the width and amplitude of APs recorded from the immature and mature synapses. By applying real APs and a series of voltage command paradigms (*i*.*e*. pseudo APs) that mimic physiological changes in the AP waveform during development, we report that the timing of synaptic transmission is particularly sensitive to the AP repolarization rate. Realistic APs mainly activate I_Ca_ in the form of off or tail currents to ensure the temporal fidelity of neurotransmission. However, due to temperature-dependent acceleration of activation and gating kinetics of VGCCs, Ca^2+^ entry more readily shifts from the repolarization to depolarization phase of wide APs near the physiological temperature, which may provide a mechanism to explain the long-standing discrepancy observed from different central synapses^7–9,15,16^.

## Results

### Presynaptic K^+^ and Na^+^ channels control the onset of I_Ca_ and EPSC

The calyx of Held synapse is an axosomatic synapse known for its speed and precision in transmitting temporal information in the sound localization pathway^19,31,35–37^. After onset of hearing at postnatal day (P) 12, this synapse undergoes rapid maturation to achieve its functionality. One of the major adaptations at the calyx is dramatic shortening of APs in both depolarization and repolarization time while the AP amplitude remains relatively stable^38^. This is due to developmental upregulation of voltage-gated Na^+^ and K^+^ conductances^39–43^. To investigate how these channels affect the timing of synaptic transmission, we first made simultaneous paired whole-cell patch clamp recordings of APs from the presynaptic terminals and of EPSC from the postsynaptic neurons in the medial nucleus of the trapezoid body (MNTB) of brainstem slices acutely obtained from immature (pre-hearing, P8-12) and mature (post-hearing, P16-20) mice. A bipolar electrode was placed onto the afferent axons to reliably evoke the APs and EPSC at 30–50% above the stimulation threshold in an all-or-none manner, confirming that each MNTB neuron is innervated at the soma by a single axon. EPSC were isolated by inhibiting GABA_A_ and glycine receptors with bicuculline (10 µM) and strychnine (1 µM), respectively. Because extracellular Mg^2+^ (1 mM) blocked N-methyl-D-aspartate (NMDA) receptors at the holding potential of −60 mV^44^, the EPSC were predominantly mediated by α-amino-3-hydroxy-5-methyl-4-isoxazolepropionic acid (AMPA) receptors^45,46^.

**Figure 1:**
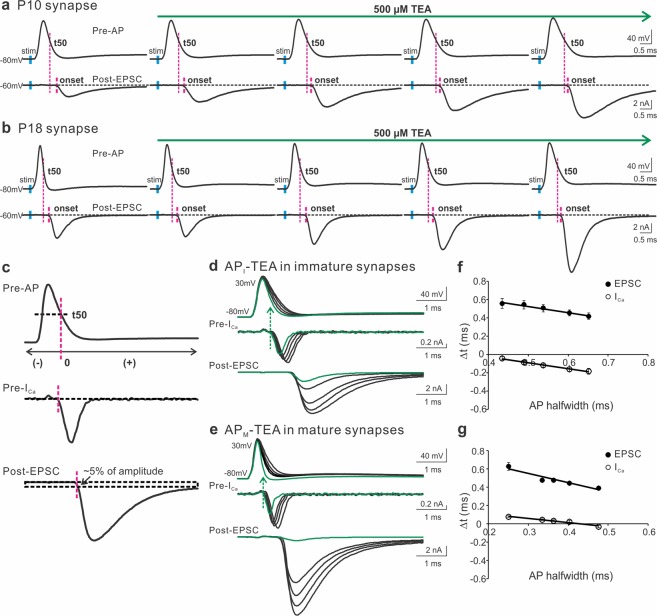
The contribution of presynaptic K^+^ channels to the onset of I_Ca_ and EPSC. (**a**,**b**) A representative AP (top panels) and EPSC (bottom panels) recorded from pre- and postsynaptic compartments of the calyx of Held synapse in response to axonal stimulation (blue bars) applied to a brain slice taken from a mouse at P10 (**a**) or P18 (**b**). TEA (500 μM) in the external solution containing 2 mM Ca^2+^ was perfused to gradually block K^+^ channels. The magenta lines indicated the onset of EPSC relative to the half decay time (t50) of APs. (**c**) t50 was defined as the repolarization time at the half-maximal amplitude of an AP and was set as time zero. Before or after t50 had negative or positive values. The onset of I_Ca_ was marked as the beginning of the inward current below the baseline. The onset of EPSC was determined by the rise within 5% of their amplitude. (**d**,**e**) Paired recordings of I_Ca_ and EPSC from immature (**d**) and mature (**e**) synapses evoked by the AP templates previously recorded from the P10 (**a**) and P18 (**b**) synapses, respectively. The currents produced by real APs before TEA exposure were highlighted in green. The extracellular solution included 1 mM Ca^2+^ to improve the quality of recordings. (**f**,**g**) Summary plots of the onset timing of I_Ca_ (empty circles) or EPSC (black circles) relative to t50 against the AP halfwidth for immature (n = 11, **f**) and mature synapses (n = 6, **g**). Solid lines were linear regressions of the data.

As we demonstrated previously^10^, APs recorded from mature nerve terminals (AP_M_ halfwidth: 0.27 ± 0.02 ms) were significantly narrower than those from immature synapses (AP_I_ halfwidth: 0.41 ± 0.01 ms). Figure 1a,b (left panels) displayed a representative AP from each group. Blocking high-threshold K^+^ channels by a low dosage of TEA (500 µM) gradually broadened the presynaptic APs with a slight effect on their amplitude, leading to a drastic increase in the size of EPSC likely due to the cooperative action of opened VGCCs during an AP in triggering transmitter release^12–14^. To determine if inhibiting K^+^ channels also changes the timing of EPSC, we inspected the temporal difference between the half decay time (repolarization time at the half-maximal amplitude, t50) of APs and the onset of EPSC. In both age groups, we noticed that TEA shortened the synaptic delay as indicated by the gaps between the two adjacent magenta lines. To quantitatively measure how broadening APs by TEA affects the timing of presynaptic Ca^2+^ influx and transmitter release, we utilized the two sets of real APs generated from the immature and mature synapses as voltage-clamp commands to simultaneously record I_Ca_ and EPSC from the pre- and postsynaptic compartments in the age-matching synapses (Fig. 1d,e). The paired recordings were performed in an extracellular solution containing 1 mM Ca^2+^ ([Ca^2+^]_e_) to improve the quality of voltage clamp by reducing the magnitude of I_Ca_, and prevent saturation of presynaptic release apparatus, as well as desensitization of postsynaptic glutamate receptors^47–49^. We here defined t50, but not the end of APs, as time zero because the membrane potential could not be effectively repolarized to the resting level in the presence of the K^+^ channel antagonist TEA (Fig. 1c). The onset of I_Ca_ was determined by the beginning of the inward current below the baseline. The start of EPSC was quantified by the rise of EPSC within ~5% of their amplitude. We carefully assured our measurements using another method, “maximum curvature”, which estimated the beginning of EPSC as the point of maximal curvature along their rise phase^50^. The estimations by the two methods were consistent (data not shown). Figure 1d,e showed that TEA mainly prolonged the repolarization time of APs and thereby augmented the size of I_Ca_ and EPSC. When we calculated the time differences (Δt) between the onset of I_Ca_ or EPSC and the half decay time of their corresponding APs, we noted that blockade of K^+^ channels precipitated an early arrival of the pre- and postsynaptic responses, independent of the developmental stages (Fig. 1f,g).

**Figure 2:**
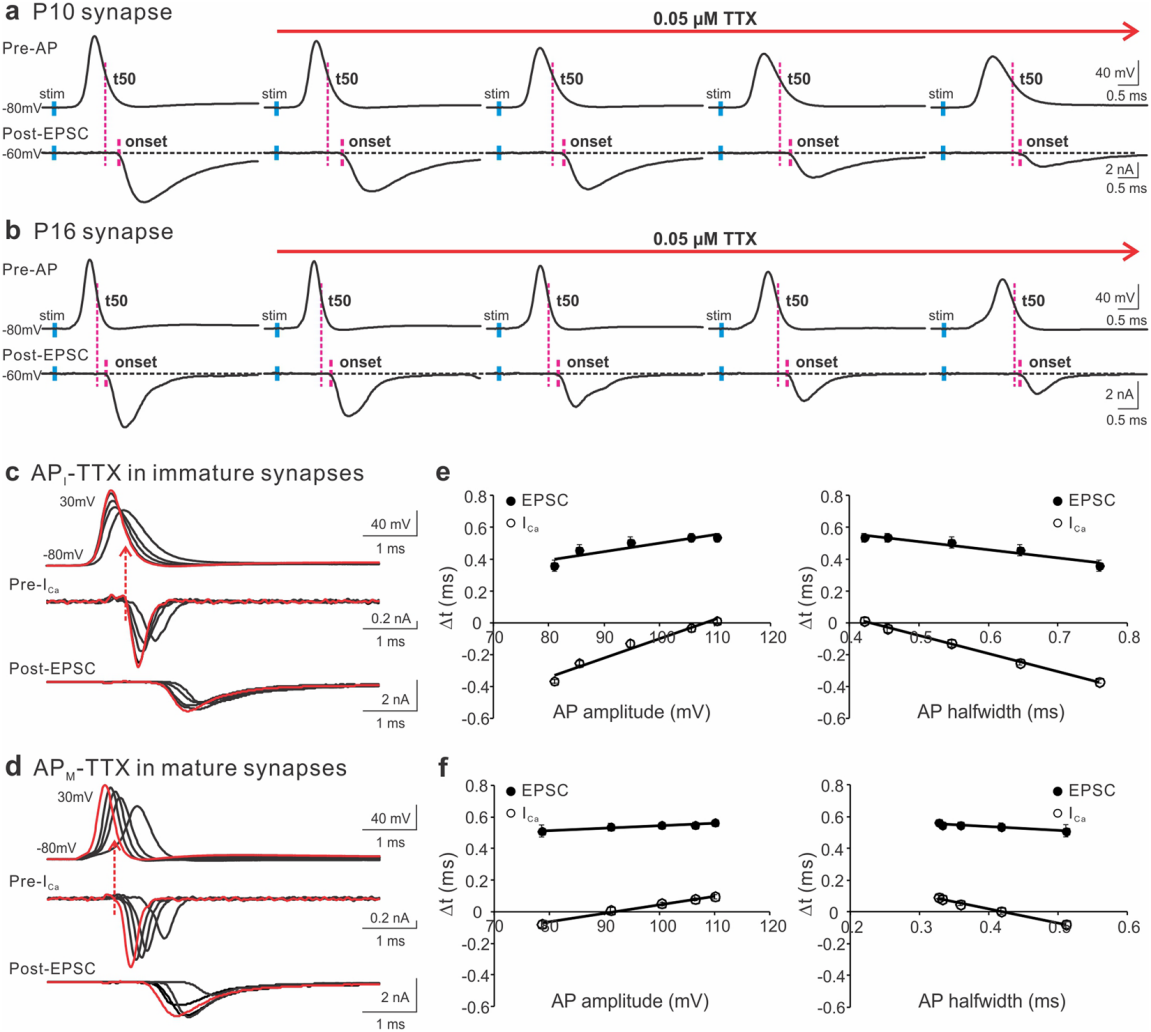
The contribution of presynaptic Na^+^ channels to the onset of I_Ca_ and EPSC. (**a**,**b**) A representative AP (top panels) and EPSC (bottom panels) recorded from pre- and postsynaptic compartments of the calyx of Held synapse in response to axonal stimulation (blue bars) applied to a brain slice taken from a mouse at P10 (**a**) or P16 (**b**). TTX (0.05 μM) in the external solution containing 2 mM Ca^2+^ was perfused to gradually block Na^+^ channels. In addition to changing the shape of APs, TTX slowed down axon conductivity by delaying AP initiation in the nerve terminal after stimulation. The magenta lines indicated the onset of EPSC relative to the half decay time (t50) of APs. (**c**,**d**) Paired recordings of I_Ca_ and EPSC from immature (**c**) and mature (**d**) synapses evoked by the AP templates previously recorded from the P10 (**a**) and P16 (**b**) synapses, respectively. The currents produced by real APs before TTX exposure were highlighted in red. The extracellular solution included 1 mM Ca^2+^ to improve the quality of recordings. (**e**,**f**) The onset timing of I_Ca_ (empty circles) or EPSC (black circles) relative to t50 were plotted against the amplitude (left panels) or halfwidth (right panels) of APs for immature (n = 10, **e**) and mature synapses (n = 6, **f**). Solid lines were linear regressions of the data.

With similar approaches, we applied TTX at a low concentration (0.05 µM) to inhibit Na^2+^ channels in the immature and mature synapses (Fig. 2a,b). As expected^51,52^, in addition to lowering the amplitude of APs, TTX extended the AP width and axonal conduction time, as shown by the delayed initiation of presynaptic APs from the axonal stimulation (short blue bars). Despite of its multifaceted impact on the AP waveform, TTX reduced the time lags in the commencement of EPSC after the peak of APs, illustrated by the two adjacent magenta lines. To bypass the confounding effect of TTX on the axon conductivity, we directly voltage-clamped the nerve terminals with the AP templates, recorded from the native synapses, to evoke presynaptic I_Ca_ and postsynaptic EPSC (Fig. 2c,d). We found that TTX altered the amplitude and width of APs by largely slowing down the AP depolarization rates, leading to a shift in the timing of I_Ca_ and EPSC. When we quantitatively correlated the take-off time of I_Ca_ and EPSC to the changes in APs, we revealed that a concurrent decrease in the AP amplitude and increase in the AP width by TTX advanced the onset of Ca^2+^ influx and vesicular release (Fig. 2e,f). Taken together, these results suggest that both presynaptic K^+^ and Na^+^ channels contribute to the timing of synaptic transmission by controlling the waveform of APs, which is highly conserved throughout development of the central synapse.

### The duration of AP repolarization but not depolarization determines the onset timing of I_Ca_

**Figure 3:**
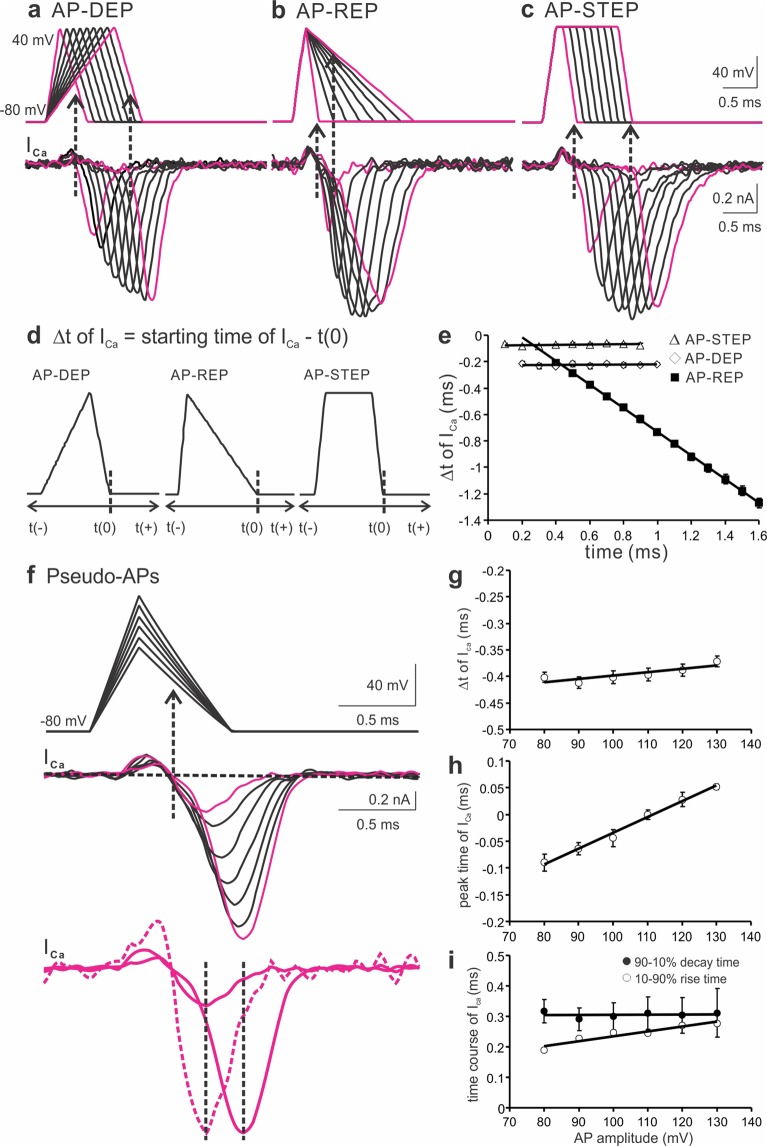
The effects of depolarization, repolarization and amplitude of an AP on the timing of presynaptic Ca^2+^ entry. (**a**–**c**) Examples of I_Ca_ (bottom panels) in response to three sets of AP-like voltage ramps (top panels) from −80 to 40 mV: AP-DEP (depolarization time from 0.2 to 1.0 ms with 0.1 ms increments, repolarization time 0.4 ms, **a**), AP-REP (depolarization time 0.2 ms, repolarization time from 0.2 to 1.6 ms with 0.2 ms increments, **b**), and AP-STEP (depolarization and repolarization time 0.2 ms, plateau duration from 0.1 to 0.9 ms with 0.1 ms increments, **c**). (**d**) Diagram showing the definition of time zero as the end of repolarization phase in the three pseudo-AP protocols. (**e**) The relative time (Δt) between time zero and the starting point of I_Ca_ was measured and plotted against the duration of depolarization (AP-DEP, open diamonds, n = 4), repolarization (AP-REP, filled squares, n = 4) and plateau step (AP-STEP, open triangles, n = 4), respectively. (**f**) I_Ca_ (middle panel) generated by a series of AP-like voltage paradigms (top panel) with increasing amplitude from 80 to 130 mV (depolarization time 0.3 ms, repolarization time 0.6 ms). In the bottom panel, the dotted line was a normalized trace of the first I_Ca_ to the last one, showing their different peak time. (**g**–**i**) Summary plots of the onset time (Δt, (**g**), peak time (**h**), 10–90% rise time (open circles, (**i**) and 90–10% decay time (filled circles, (**i**) of I_Ca_ against the AP amplitude (n = 5). Solid lines were linear regression fits with distinct slopes for the onset time (0.0006), peak time (0.0029), 10–90% rise time (0.0016) and 90-10% decay time (0.00006). Recordings were made from P8-12 synapses in 1 mM [Ca^2+^]_e_ in this and following figures unless otherwise specified.

As voltage-gated Na^+^ and K^+^ conductances are interlocked to the membrane potential, pharmacological inhibition of these channels makes it difficult to separate the roles of the amplitude and kinetics of APs in regulating the timing of presynaptic Ca^2+^ influx (Figs 1 and 2). To circumvent the complicate properties of Na^+^ and K^+^ channel blockers, we designed three sets of AP-like voltage clamp commands with the same amplitude, *i*.*e*. AP-DEP, AP-REP and AP-STEP, which referred to the specific changes in the depolarization, repolarization and plateau duration of APs, respectively (Fig. 3a–c, top panels). In response to the three paradigms, the amplitude of I_Ca_ increased as the AP width was broadened and eventually saturated, as previously described^10^. When the time interval (Δt) between the end point of AP repolarization phase (time zero, t(0)) and the start point of I_Ca_ was quantified (Fig. 3d), we found that I_Ca_ were essentially tail currents for AP-DEP and AP-STEP paradigms (Fig. 3a,c). This indicated that Ca^2+^ entry took place near the end of the repolarization phase of APs with a Δt of ~0.2 ms or less, independent of the depolarization or step duration (Fig. 3e). However, I_Ca_ evoked by the AP-REP protocol were initially tail currents when the repolarization time was short (Fig. 3b). As the repolarization time was prolonged, the onset of I_Ca_ advanced towards the early part of AP repolarization phase and appeared as typical off currents, in parallel with changes in the amplitude and kinetics of I_Ca_. When Δt was plotted against the duration of AP repolarization, Δt linearly shifted to the negative values as the repolarization was extended (Fig. 3e). For instance, Δt changed from −0.21 ± 0.01 ms for 0.4 ms of repolarization time to −1.27 ± 0.04 ms for 1.6 ms of repolarization time. These results demonstrate that the timing of Ca^2+^ influx into the nerve terminal is dependent on the time course of AP repolarization but not depolarization, consistent with the effect of TEA on the real AP-driven I_Ca_ (Fig. 1).

### The amplitude of APs affects the peak time but not onset of I_Ca_

Among different central synapses, presynaptic APs vary in the amplitude, which may affect the driving force for Ca^2+^ inflow via VGCCs and hence the kinetics and timing of I_Ca_^10^. By applying a series of pseudo-APs with the same depolarization and repolarization time yet varied amplitude, ranging from 80 to 130 mV (Fig. 3f, top panel) to evoke I_Ca_, we found that increasing the AP amplitude raised the size of I_Ca_ (from 0.41 ± 0.06 nA for 80 mV to 1.39 ± 0.15 nA for 130 mV of APs) and delayed the peak time of I_Ca_ (from −0.09 ± 0.01 ms for 80 mV to 0.05 ± 0.005 ms for 130 mV of APs, Fig. 3h), with a marginal effect on the onset timing of I_Ca_ (−0.40 ± 0.01 ms and −0.37 ± 0.01 ms for 80 mV and 130 mV of APs, respectively, Fig. 3g). Further analysis on the kinetics of evoked I_Ca_ showed that the rise time of I_Ca_ was slowed (from 0.19 ± 0.003 ms for 80 mV to 0.28 ± 0.01 ms for 130 mV of APs), while the decay time of I_Ca_ remained the same (0.32 ± 0.04 ms for 80 mV *vs* 0.31 ± 0.08 ms for 130 mV of APs, Fig. 3i), indicating that the AP amplitude influenced the activation but not deactivation time course of VGCCs during an AP. Our results suggest that the onset timing of I_Ca_ is insensitive to changes in the AP amplitude, which however exerts a significant impact on the magnitude of I_Ca_, likely by increasing the number and open probability of recruited VGCCs as well as the driving force for Ca^2+^ influx. The observation is not in conflict with the effect of TTX on the timing of I_Ca_ because TTX broadens APs in parallel to lowering their amplitude (Fig. 2).

### The timing of I_Ca_ is independent of extracellular Ca^2+^ concentration

**Figure 4:**
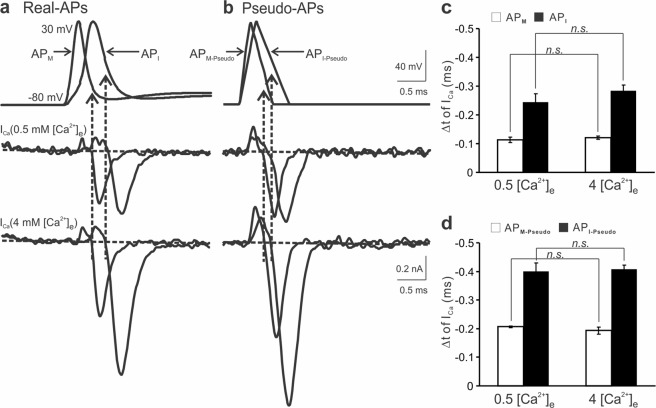
Timing of presynaptic Ca^2+^ influx in different extracellular Ca^2+^ concentrations. (**a**,**b**) Recordings of I_Ca_ in 0.5 mM (middle panels) or 4 mM [Ca^2+^]_e_ (bottom panels) from the same synapses in response to real APs (a, top panel) previously obtained from a P11 (AP_I_, −80 to 30 mV, depolarization and repolarization time: 0.46 and 0.8 ms, halfwidth 0.41 ms) or P17 calyx (AP_M_, −80 to 30 mV, depolarization and repolarization time: 0.2 and 0.5 ms, halfwidth 0.27 ms) and voltage ramps with similar waveform (AP_I-Pseudo_, −80 to 30 mV, depolarization and repolarization time: 0.3 and 0.6 ms, halfwidth 0.45 ms; AP_M-Pseudo_, −80 to 30 mV, depolarization and repolarization time: 0.2 and 0.4 ms, halfwidth 0.3 ms, (b, top panel)^10^. (**c**,**d**) Summaries of the relative time for presynaptic Ca^2+^ entry produced by real (**c**) or pseudo APs (**d**) in low and high [Ca^2+^]_e_ (n = 5).

Having used pseudo-APs to examine the effects of various components of an AP on I_Ca_, we next investigated how the waveform of physiologically relevant APs influences the timing of I_Ca_. As illustrated in Fig. 4a, we employed previously obtained two representative APs from immature (AP_I_) and mature (AP_M_) calyces^10^ as templates to evoke I_Ca_. Conjointly, two pseudo-APs with the comparable halfwidth were applied to the same terminals (Fig. 4b). In all cases, the timing of inward I_Ca_ fell in the repolarization phase with their onset shifting forward to the peak for wider APs. This shift was expected as demonstrated in Fig. 3, in which prolonged repolarization duration led to an early activation of I_Ca_. Raising [Ca^2+^]_e_ markedly increased the amplitude of I_Ca_ evoked by real APs (AP_M_: 0.35 ± 0.03 nA in 0.5 mM and 0.70 ± 0.08 nA in 4 mM [Ca^2+^]_e_, AP_I_: 0.48 ± 0.01 nA in 0.5 mM and 1.27 ± 0.13 nA in 4 mM [Ca^2+^]_e_) and pseudo APs (AP_M-Pseudo_: 0.42 ± 0.07 nA in 0.5 mM and 0.70 ± 0.12 nA in 4 mM [Ca^2+^]_e_, AP_I-Pseudo_: 0.50 ± 0.05 nA in 0.5 mM and 1.29 ± 0.14 nA in 4 mM [Ca^2+^]_e_) (Fig. 4a,b, middle and bottom panels). But it did not affect the activation time of I_Ca_ produced by real APs (AP_M_: −0.11 ± 0.01 ms in 0.5 mM and −0.12 ± 0.01 ms in 4 mM [Ca^2+^]_e_, AP_I_: −0.24 ± 0.03 ms in 0.5 mM and −0.28 ± 0.02 ms in 4 mM [Ca^2+^]_e_, Fig. 4c) or pseudo APs (AP_M-Pseudo_: −0.21 ± 0.003 ms in 0.5 mM and −0.19 ± 0.01 ms in 4 mM [Ca^2+^]_e_, AP_I-Pseudo_: −0.40 ± 0.03 ms in 0.5 mM and −0.41 ± 0.01 ms in 4 mM [Ca^2+^]_e_, Fig. 4d). Because APs at the calyx of Held nerve terminals overshoot to +30 mV where the open probability of VGCCs has approached the maximum, these data suggest that changing the driving force for Ca^2+^ influx by altering [Ca^2+^]_e_ has little effect on the timing of I_Ca_, which remain as tail/off currents under the experimental conditions.

### Developmental regulation of the timing of I_Ca_ and EPSC

**Figure 5:**
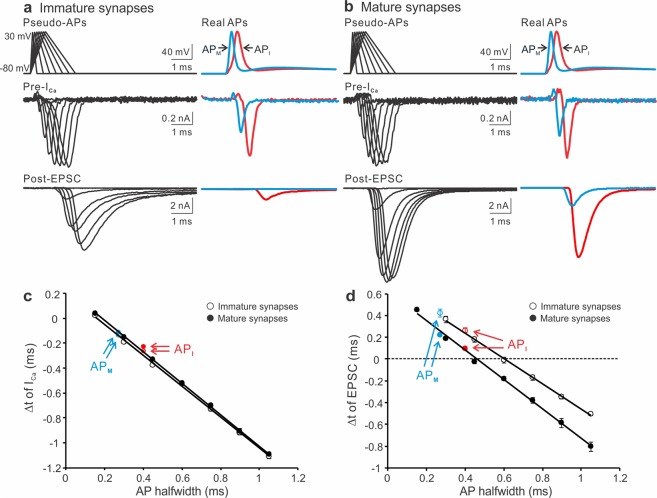
Developmental changes in the commencement of I_Ca_ and EPSC. (**a**,**b**) Paired recordings of I_Ca_ (middle panels) and EPSC (bottom panels) evoked by pseudo- (−80 to 30 mV, depolarization time from 0.1 to 0.7 ms with 0.1 ms increments, repolarization time from 0.2 to 1.4 ms with 0.2 ms increments, left panels) and real APs (described in Fig. 4, right panels) from P8-12 (**a**) or P16-20 (**b**) synapses. I_Ca_ and EPSC produced by the realistic immature AP_I_ or mature AP_M_ were labelled in red or blue, respectively. (**c**,**d**) The time intervals (Δt) between the end of AP repolarization phase and the start of I_Ca_ (**c**) or EPSC (**d**) were plotted against the AP halfwidth for the immature (open circles, n = 7) and mature (filled circles, n = 8) groups. Solid lines were linear regressions of the data.

Because depolarization and repolarization phases shorten simultaneously during early postnatal development at the calyx of Held synapse^38^, we next studied the impacts of changing both phases on the timing of I_Ca_ and EPSC. To this end, we designed a set of pseudo-APs, which had the same amplitude (*i*.*e*. −80 to +30 mV, 110 mV) but a wide spectrum of depolarization and repolarization rates to mimic developmental changes in the waveform of APs (Fig. 5a,b). In parallel, we used the two typical real AP_I_ and AP_M_ as voltage-clamp commands to evoke I_Ca_ and EPSC from either the immature or the mature synapses. In both groups, the magnitude of I_Ca_ and EPSC increased as the depolarization and repolarization phases of pseudo-APs were prolonged. To quantify the timing of I_Ca_ and EPSC, we measured Δt between the start of the inward I_Ca_ or EPSC and the end of the AP repolarization phase, and plotted against the halfwidth of APs. Figure 5c,d exhibited strong correlations between the two parameters giving similar slope values for the two age groups (I_Ca_: −1.24 for immature and −1.26 for mature synapses; EPSC: −1.16 for immature and −1.35 for mature synapses), suggesting that the timing of Ca^2+^ entry and transmitter release is highly sensitive to the AP width. Although developmentally the VGCCs that mediate vesicular fusion in the calyces switch from a mixture of P/Q-, N- and R-types to predominantly P/Q-type^18,53^, the timing of Ca^2+^ inflow through these channels remained constant over maturation (Fig. 5c), reinforcing that the temporal control of presynaptic Ca^2+^ influx is unaffected by the different subtypes of VGCCs^17–19^. By contrast, the onset of EPSC at the mature synapses was significantly faster than that at the immature ones at any given AP halfwidth (Fig. 5d). This is likely attributed to the developmental tightening of spatial distance between VGCCs and release sites^47,54^.

The onset of I_Ca_ and EPSC produced by real APs followed the correlations and fell within the latency range evoked by narrow pseudo-APs, with AP_M_ generating longer Δt of EPSC than AP_I_. It is of physiological importance to maintain sufficient synaptic delay for ensuring information flow in one direction and for coordinating network activity^55^. Had AP narrowing not took place in the mature synapses, AP_I_ would decrease the Δt for EPSC by a fold (0.22 ± 0.02 ms for AP_M_ and 0.10 ± 0.01 ms for AP_I_), jeopardizing the information transfer from pre- to postsynaptic neurons, particularly when the release efficacy has been enhanced by tight spatial coupling to VGCCs^47,54^.

### Temperature accelerates voltage-dependent activation of I_Ca_ by APs

**Figure 6:**
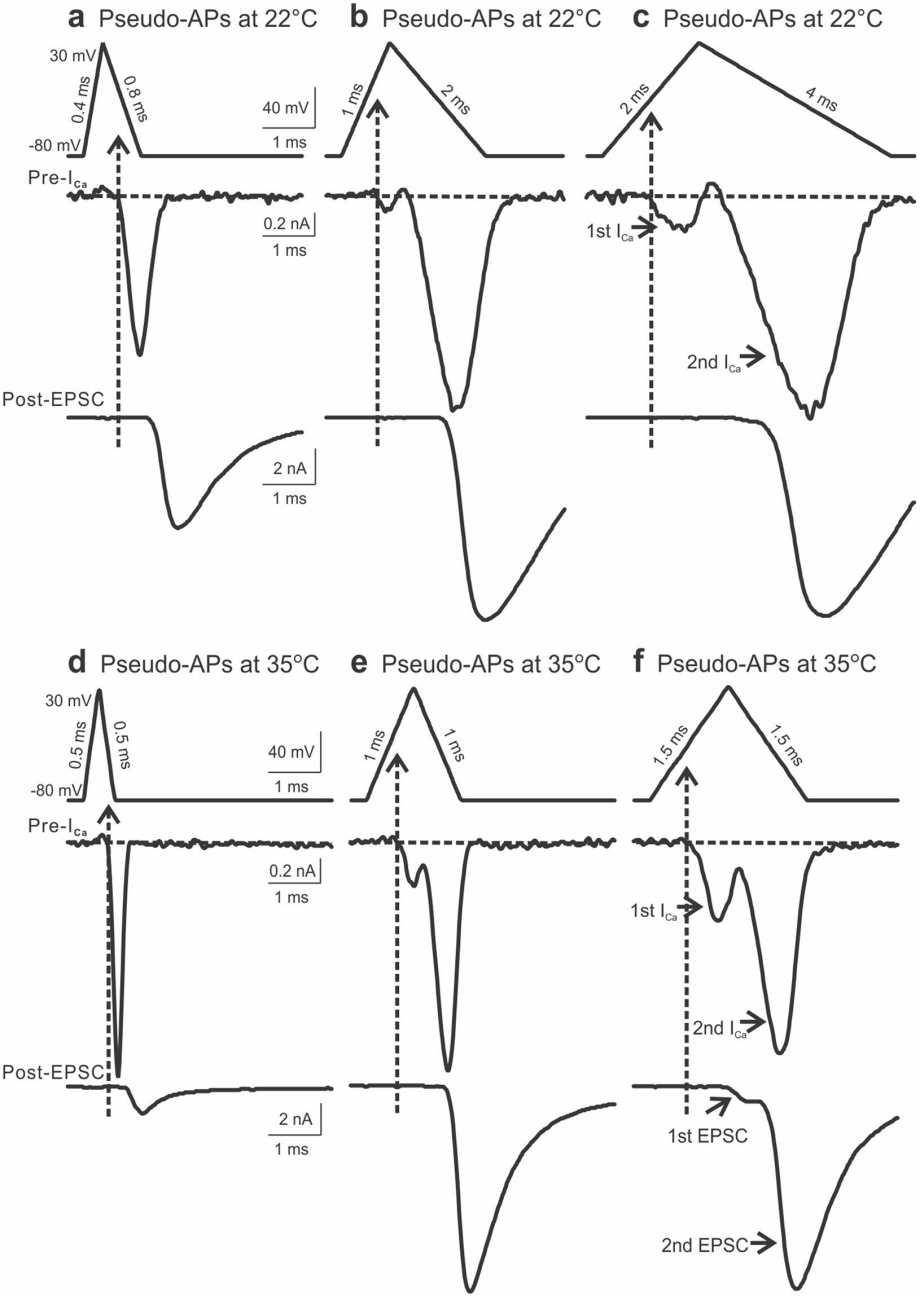
Dual-peak I_Ca_ and EPSC evoked by prolonged APs. (**a**–**c**) Examples of paired recordings of I_Ca_ (middle panels) and EPSC (bottom panels) evoked by pseudo APs (**a** depolarization 0.4 ms and repolarization 0.8 ms, **b** depolarization 1 ms and repolarization 2 ms, **c** depolarization 2 ms and repolarization 4 ms) with the same amplitude of 110 mV at 22 °C. (**d**–**f**) Similar recordings of I_Ca_ (middle panels) and EPSC (bottom panels) evoked by pseudo APs (**d** depolarization and repolarization time 0.5 ms, **e** depolarization and repolarization time 1.0 ms, **f** depolarization and repolarization time 1.5 ms) with the same amplitude of 110 mV at 35 °C. Note the appearance of dual Ca^2+^ transients, the first of which occurred during the depolarization phase of wide APs (**b**,**c**,**e**,**f**). This was more prominent at a higher temperature.

Thus far, our results are in line with previous work from the calyx of Held synapse^8^, showing that Ca^2+^ influx starts during the repolarization phase. However, at the cerebellar parallel fiber-stellate cell synapse, the timing of Ca^2+^ influx was highly sensitive to temperature^7^. At the physiological temperature, Ca^2+^ entry could occur as early as in the AP depolarization phase, registered as on currents, in contrast to the conclusion from the calyx of Held synapse that the timing of Ca^2+^ influx is independent of the experimental temperature^8^. To explore potential explanations for such an apparent discrepancy, we delivered a series of pseudo-APs with varied width to the calyces and performed paired recordings of I_Ca_ and EPSC at room temperature (22 °C) and near physiological temperature (35 °C). Figure 6 displayed three sets of such recordings evoked by pseudo-APs with incremental changes in the depolarization time and repolarization time for both temperatures. Noticeably, the narrowest AP produced an I_Ca_ with its activation onset in the repolarization phase (Fig. 6a,d), but dual-peak I_Ca_ appeared when evoked by the other two wider APs with a small inward current preceding the main tail/off current (Fig. 6b,c,e,f). This was more prominent at the higher temperature. In fact, as shown in Fig. 6f, the initial inward I_Ca_ was sufficient to induce glutamate release, generating a double-component compound EPSC. This observation indicated that at the physiological temperature, Ca^2+^ could enter the nerve terminal during the AP depolarization phase and trigger transmitter release. In the case where the first Ca^2+^ transient did not trigger release, it was likely because such a small Ca^2+^ influx had failed to reach the threshold of local Ca^2+^ domains for vesicular fusion, particularly in the immature synapses where VGCCs are situated far away from synaptic vesicles^47,54^.

**Figure 7:**
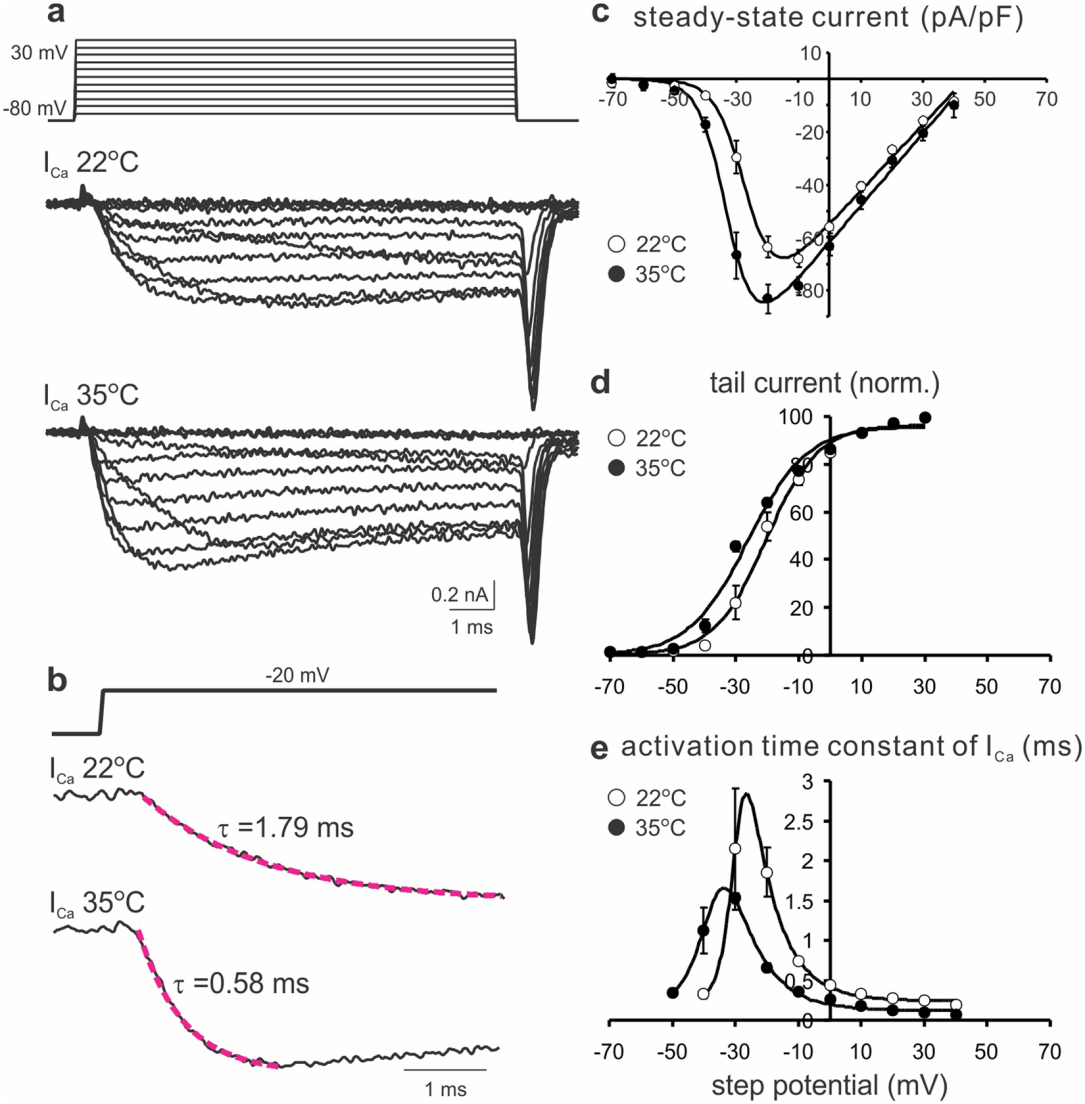
Temperature effects on the properties of VGCCs. (**a**) Example traces of I_Ca_ elicited by voltage steps (top panel) of 10 ms duration from −70 to + 30 mV in 10 mV increments at 22 °C (middle panel) and 35 °C (bottom panel). (**b**) The activation phase of I_Ca_ evoked by a voltage step to −20 mV (top panel) at 22 °C (middle panel) and 35 °C (bottom panel) was fitted by a single-exponential function (magenta lines). Time constants were given. (**c**) Current-voltage relationships of I_Ca_ recorded at 22 °C (open circles, n = 5) and 35 °C (filled circles, n = 5) were described by an equation: *f* (V) = g*(V-V_R_)/(1 + exp(−0.03937*z*(V-V_1/2_)))^62^, where the half-maximal activation potential V_1/2_ were −26.4 and −32.6 mV for 22 °C and 35 °C, respectively. (**d**) Tail currents were normalized to the maximal value for each nerve terminal and plotted against step potentials. The activation curves (solid lines) were generated by fitting with a Boltzmann function *f* (V) = Imax/{1 + Exp[(V_1/2_ − V)/Vc]} + C. The V_1/2_ values were −20.4 and −26.2 mV and the slope factors (Vc) were 8.2 and 9.4 for 22 °C and 35 °C, respectively. (**e**) The activation time constants of I_Ca_ (as measured in **b** for different voltages) were plotted for 22 °C (open circles, n = 5) and 35 °C (filled circles, n = 5). Solid lines were fits with an equation *f* (V) = 1/{a_0_ Exp[V/K_1_] + b_0_ Exp[−V/K_2_]} + C, where K_1_ and K_2_ values were 8.85 and 2.74 for 22 °C, and 9.65 and 5.50 for 35 °C.

To test if the early onset of I_Ca_ was resulted from accelerated gating kinetics of VGCCs by high temperature, we compared the properties of I_Ca_ evoked by voltage steps (−70 to +30 mV, 10 mV increment, 10 ms long) from calyces at 22 °C and 35 °C (Fig. 7a). The current-voltage relationships revealed a significant increase in the maximal current density of I_Ca_ (−67.89 ± 3.29 pA/pF at 22 °C and −83.15 ± 5.36 pA/pF at 35 °C) and a left shift in their voltage-dependence at 35 °C as compared to 22 °C (Fig. 7a,c). The half-maximal activation potential (V_1/2_) for 22 °C and 35 °C were estimated as −26.4 and −32.6 mV, respectively. The amplitude of tail currents was normalized to the maximum and plotted against voltage steps (Fig. 7d). The curves were well fitted with a Boltzmann function, giving V_1/2_ values of −20.4 and −26.2 mV, and slope factors of 8.2 and 9.4 mV for 22 °C and 35 °C, respectively. These observations suggest that at 35 °C VGCCs are activated at more negative potentials and have a higher open probability than at 22 °C. Furthermore, the activation time constants (τ) were measured by fitting an exponential function to the onset segment of I_Ca_ in response to different step potentials (Fig. 7b). Figure 7e demonstrated that the time constants were strongly voltage-dependent and significantly shortened at 35 °C when compared to those at 22 °C at the corresponding potentials. For example, τ at −20 mV displayed about three-fold difference, being 1.85 ± 0.31 ms at 22 °C and 0.66 ± 0.05 ms at 35 °C. Therefore, it can be rationalized that at the physiological temperature a substantial fraction of VGCCs are activated during the depolarization phase of a pseudo-AP due to a left-shifted activation threshold and faster activation kinetics. However, as the depolarization voltage (−80 to + 30 mV) approaches the reversal potential, the first component of I_Ca_ only appears as a brief transient because of the diminishing driving force for Ca^2+^ inflow and then the second peak of I_Ca_ is generated as the driving force for Ca^2+^ reestablishes during the repolarization phase (+30 to −80 mV). Taken together, our results suggest that at 35 °C a combination of a more negative activation threshold and accelerated activation kinetics of VGCCs can lead to an early onset of Ca^2+^ entry into the nerve terminal during the AP depolarization phase, only if the AP waveform is sufficiently wide as seen in certain slow-spiking neurons^20^.

### Physiological impact of temperature-dependent changes in APs on the timing of I_Ca_ and EPSC

**Figure 8:**
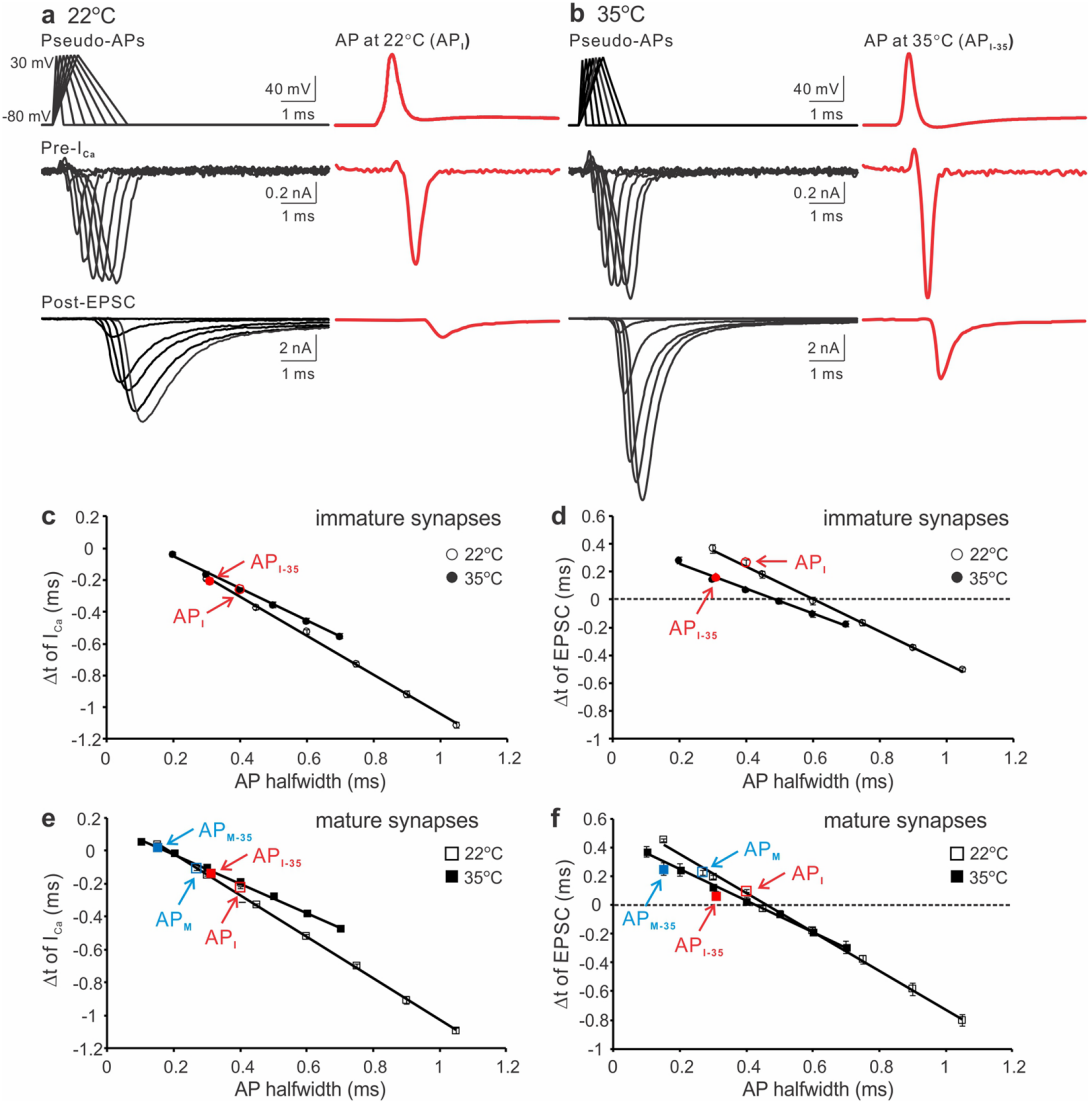
Temperature dependence of the onset timing of I_Ca_ and EPSC. (**a**,**b**) Simultaneous recordings of I_Ca_ (middle panels) and EPSC (bottom panels) produced by pseudo- (left panels) and real APs (right panels) from P8-12 synapses at 22 °C (**a**) or 35 °C (**b**). Pseudo APs at 22 °C (**a**, top left): −80 to 30 mV, depolarization time from 0.1 to 0.7 ms with 0.1 ms increments, repolarization time from 0.2 to 1.4 ms with 0.2 ms increments. Pseudo APs at 35 °C (**b**, top left): −80 to 30 mV, depolarization time from 0.1 to 0.7 ms with 0.1 ms increments, repolarization time from 0.1 to 0.7 ms with 0.1 ms increments. The real AP recorded at 22 °C (AP_I_, **a** top right) is the same as described in Fig. 4. The real AP recorded at 35 °C (AP_I-35_, **b** top right) from a P11 calyx: −80 to 30 mV, depolarization and repolarization time 0.4 and 0.5 ms respectively, halfwidth 0.3 ms. (**c**,**d**) The time differences (Δt) between the start of I_Ca_ (**c**) or EPSC (**d**) and the end of AP repolarization phase were summarized for 22 °C (open circles, n = 7) and 35 °C (filled circles, n = 6). (**e**,**f**) Summary plots of the onset timing of I_Ca_ (**e**) and EPSC (**f**) evoked by the same pseudo- and real AP templates in P16-20 synapses at 22 °C (empty squares, n = 8) or 35 °C (black squares, n = 5).

Raising temperature shortens APs in both depolarization and repolarization phases^7,10,38^. To study how the alterations play a role in controlling the initiation of I_Ca_ and EPSC, we digitally generated two sets of pseudo-APs based on the real APs recorded from the calyx of Held synapses at 22 °C and 35 °C^10^. The APs had the same amplitude (110 mV) but different depolarization and repolarization durations to simulate the developmental modifications of APs at the two temperatures (Fig. 8a,b, top left panels). For instance, at 22 °C the repolarization time was twice as long as the depolarization time of an AP whereas at 35 °C the length of depolarization or repolarization for an AP was equal. Meanwhile, representative APs recorded from P8-12 calyces at both temperatures were used as voltage-clamp templates (Fig. 8a,b, top right panels)^10^. Using the same synapses, we simultaneously recorded I_Ca_ (middle panels) and EPSC (bottom panels) evoked by the pseudo- and real-APs (Fig. 8a,b). In both cases, they increased as the depolarization and repolarization phases of pseudo-APs were expanded. However, I_Ca_ and EPSC at 35 °C displayed much larger size and faster kinetics than those at 22 °C. To quantify their onset timing, we again measured the time intervals (Δt) between the start of the inward I_Ca_ or EPSC and the end of the AP repolarization phase. Figure 8c,d showed that Δt linearly correlated to the AP halfwidth at both temperatures with distinct slope factors (I_Ca_: −1.23 at 22 °C and −1.01 at 35 °C; EPSC: −1.16 at 22 °C and −0.89 at 35 °C). The onset of I_Ca_ and EPSC driven by real APs was comparable to that evoked by pseudo-APs with the similar waveform. The AP obtained at a higher temperature (AP_I-35_) was briefer and produced shorter Δt (22 °C: −0.26 ± 0.004 ms for I_Ca_ and 0.26 ± 0.02 ms for EPSC; 35 °C: −0.20 ± 0.01 ms for I_Ca_ and 0.16 ± 0.02 ms for EPSC). When we did the same recordings by voltage clamping the P16-20 synapses with the pseudo- and realistic immature and mature APs acquired at the two temperatures, we observed that the AP waveform and temperature dependent regulation on the timing of I_Ca_ and EPSC was consistent throughout development (Fig. 8e,f). Collectively, these results demonstrate that acceleration in depolarization and repolarization phases ensures rapid I_Ca_ as tail/off transients, potentially minimizing temporal jitter of Ca^2+^ influx and transmitter release to enhance the fidelity of synaptic transmission.

## Discussion

In this study, we combine pharmacological and biophysical manipulations of the presynaptic AP waveform to elucidate the effects of changing AP depolarization, repolarization and amplitude on the onset of I_Ca_ and EPSC in the developing calyx of Held synapse. Our results suggest that the timing of synaptic transmission is best preserved in the form of Ca^2+^ tail currents under physiological conditions, which is important for high-fidelity neurotransmission with precise temporal control of presynaptic release and postsynaptic response.

Although the magnitude of I_Ca_ evoked by real and pseudo-APs increases with prolonged depolarization and repolarization periods, the onset timing of I_Ca_ is exclusively determined by the repolarization rates (Fig. 3). The amplitude of APs and [Ca^2+^]_e_ also have profound impact on the size and peak time of I_Ca_ but not their onset timing (Figs 3 and 4). Ca^2+^ influx evoked by physiological APs begins during or near the end of the repolarization phase referred as off or tail currents (Figs 1, 2, 4, 5 and 8). When the AP waveform broadens, as illustrated by the sets of pseudo APs, the initiation of I_Ca_ and EPSC shifts towards the depolarization phase with strong linear correlations to the AP width (Figs 5 and 6). This is mostly noticeable when the activation and gating kinetics of VGCCs speed up near the physiological temperature, leading to dual-component I_Ca_ and EPSC (Figs 6 and 7). In contrast to a power relationship between the integral of I_Ca_ and that of EPSC^10,54^, the onset timing for both I_Ca_ and EPSC follows a linear relationship with the AP width, separated by a virtually constant time interval (Figs 1 and 2). This indicates that downstream Ca^2+^-dependent fusion, independent of variations in the AP waveform, tightly controls the synaptic delay (between I_Ca_ and EPSC).

In the cerebellar parallel fiber-stellate cell synapse, Ca^2+^ entry moves to the AP depolarization phase in a temperature-dependent manner^7^. Although the calyx of Held synapse may preserve the timing of Ca^2+^ influx in a different manner, our observation that I_Ca_ can be activated during the depolarization phase of wide APs at 35 °C (Fig. 6) provides a proof of principle for such an early Ca^2+^ entry in the parallel fiber boutons, where APs could last for several milliseconds. We interpret that a more negative activation threshold and drastically accelerated activation rate of VGCCs at the physiological temperature likely account for the early onset of I_Ca_ (Fig. 7). Our experimental evidence also validates several theoretical predictions made by computer simulations in which increasing gating rate of VGCCs would alter the amplitude, kinetics and timing of I_Ca_^7–9,15,16^, including the appearance of dual-component Ca^2+^ transients^16^. Given that central synapses may employ heterogeneous arrays of voltage-gated conductance to generate diverse AP waveforms and multiple types of VGCCs to mediate Ca^2+^-dependent vesicular release, it is conceivable that different ways of controlling the timing of Ca^2+^ entry may provide diverse venues for synapses to serve distinct computational functions. Our results may help reconcile the opposing views on the effect of spike broadening on the commencement of presynaptic I_Ca_ at different synapses^4,8,9,16,56,57^.

In any central synapse, the relationship between the amount of Ca^2+^ influx and quantal output can be described by a power function in the form of EPSC ∝ [I_Ca_]^*m*^, where *m* denotes Ca^2+^ cooperativity^58^. We have previously demonstrated that AP narrowing is a highly effective adaptation for the calyx to reduce the I_Ca_ integral^10^, while developmental maturation (and high temperature) strengthens the efficacy of coupling Ca^2+^ to vesicular fusion as manifested by a lower *m* value^47,54^. These counteracting factors converge to limit the number of synaptic vesicles released per AP or release probability (*P*_*r*_) to conserve the readily releasable pool. The timing constraints of I_Ca_ and EPSC defined by a variety of pseudo- and real APs in this study further conclude that narrow APs generate short Ca^2+^ transients as tail currents and reliable latency in transmitter release to preserve the timing of inputs and reduce their jitters. During development I_Ca_ appears as an off current in the immature synapses, but as maturation progresses it becomes a tail current, reminiscent of the behavior of I_Ca_ evoked by an AP at the squid giant synapse^3^. Such a transformation along with other concurrent biophysical and morphological reorganizations in the active zone is important for establishment of high-fidelity neurotransmission required for sound localization at this synapse^33,37,59,60^.

## Methods

### Slice preparation

Mice were housed in the facility accredited by the Association for the Assessment and Accreditation of Laboratory Animal Care (AAALAC) and all the experiments were performed according to protocols approved by Institutional Animal Care and Use Committee (IACUC) and Institutional Biosafety Committee (IBC) of University of Minnesota. Brainstem slices were prepared from CD1xC57 hybrid mice at postnatal day (P) 8-20, as previously described^61^. Both genders were included. Following decapitation with a small guillotine, brains were immersed and dissected in semi-frozen artificial cerebral spinal fluid (ACSF) containing (in mM): NaCl (125), KCl (2.5), glucose (10), NaH_2_PO_4_ (1.25), Na-pyruvate (2), myo-inositol (3), ascorbic acid (0.5), NaHCO_3_ (26), MgCl_2_ (1), and CaCl_2_ (2) at a pH of 7.3 when oxygenated (95% O_2_ and 5% CO_2_). Transverse slices of the auditory brainstem containing the medial nucleus of the trapezoid body (MNTB) were cut at a thickness of 150–250 μm using a microtome (Leica VT1200 S) and incubated at 37 °C for one hour prior to experimentation. For current-clamp recordings of real APs, thicker slices (250 μm) were used to preserve afferent axons. For paired voltage-clamp recordings, thinner slices (150–200 μm) were prepared with a slight angle (~15° tilt away from the coronal plane) during slicing to minimize presynaptic axon length and space-clamp errors. Most experiments were performed at room temperature (~22 °C) except for several subsets of experiments in Figs 6–8, which were performed at 35 °C using an in-line heater with a feedback thermistor (Warner TC-324B).

### Electrophysiology

All recordings were acquired at a filtering frequency of 4 kHz with a dual-channel amplifier (MultiClamp 700B, Molecular Devices) and digitized at a sampling rate of 50 kHz with Digidata 1550B (Molecular Devices). ACSF was supplemented with bicuculline (10 μM) and strychnine (1 μM) to block inhibitory inputs during recording. To record presynaptic Ca^2+^ currents, tetrodotoxin (TTX, 0.5–1 μM), tetraethylammonium (TEA, 10 mM) and 4-aminopyridine (0.3 mM) were added to block Na^+^ and K^+^ channels while extracellular Ca^2+^ concentration ([Ca^2+^]_e_) was set at 1 mM. Patch electrodes typically had resistances of 4–6 MΩ and 2.5–3 MΩ for presynaptic and postsynaptic recordings, respectively. For paired voltage-clamp recordings, presynaptic and postsynaptic series resistances were <10 MΩ and <5 MΩ respectively and compensated to 90%. Recordings not reaching initial GΩ seal or holding currents higher than 300 pA were omitted. The criteria for rigorous control over the quality and stability of voltage clamp of I_Ca_ and EPSC evoked by APs were addressed in details before^10^ and were applied in this study. Intracellular solution for recording I_Ca_ contained (in mM): CsCl (110), HEPES (40), EGTA (0.5), MgCl_2_ (1), ATP-Na (2), GTP-Na (0.5), Phosphocreatine (12), TEA (20) and K-glutamate (3) (pH adjusted to 7.3 with CsOH). Intracellular solution for recording EPSC contained (in mM): K-gluconate (97.5), CsCl (32.5), EGTA (5), HEPES (10), MgCl_2_ (1), TEA (30) and lidocaine N-Ethyl Bromide (QX314, 3) (pH adjusted to 7.2 with KOH). The holding potential was −80 mV for presynaptic terminals and −60 mV for postsynaptic neurons. I_Ca_ and EPSC were evoked by various voltage commands described in the figure legend. Leak subtraction was done with the on-line P/4 protocol. The reliability of the P/4 protocol was previously verified by the author^28^. For experiments where real APs were used as presynaptic voltage commands, we first recorded APs from calyces in the current-clamp configuration by stimulating afferent axon fibers using a bipolar platinum electrode. Pipettes for these experiments were filled with an intracellular solution containing (in mM) K-gluconate (97.5), KCl (32.5), EGTA (0.5), HEPES (40), MgCl_2_ (1), ATP-Na (2) and GTP-Na (0.5) (pH adjusted to 7.3 with KOH). After manually removing stimulation artifacts preceding the APs, the digitized values were generated as voltage command templates and fed back into the amplifier as stimulation files (Axon Text File) through pClampex 10 at the same frequency as their acquisition (50 kHz). Details for the real and pseudo APs were given in the figure legends. Reagents were purchased from Sigma, Tocris and Alomone Labs.

### Data analysis

Data were analyzed off-line by pCLAMP 10 software package (Molecular Devices) and Excel (Microsoft). For analyses in Fig. 7, curve fittings were done by Clampfit (Molecular Devices), and equations were given in the figure legend. For other correlation analyses, least-squares linear regression was performed using Excel (Microsoft). Statistical significance was defined by two-sample unpaired Student’s *t*-tests assuming unequal variances with a p-value cut-off <0.05. Data were expressed as the mean ± standard error from a population of synapses (n).

## Data Availability

All the data are available upon request.
